# Histone Variant H2A.J Marks Persistent DNA Damage and Triggers the Secretory Phenotype in Radiation-Induced Senescence

**DOI:** 10.3390/ijms21239130

**Published:** 2020-11-30

**Authors:** Anna Isermann, Carl Mann, Claudia E. Rübe

**Affiliations:** 1 Department of Radiation Oncology, Saarland University Hospital, Kirrbergerstrasse Building 6.5, 66421 Homburg/Saar, Germany; anna.isermann@uks.eu; 2 Institute for Integrative Biology of the Cell (I2BC), CEA, CNRS, Université Paris-Saclay, 91198 Gif-sur-Yvette, France; Carl.MANN@cea.fr

**Keywords:** histone variant H2A.J, radiation-induced senescence, senescence-associated heterochromatin foci (SAHF), DNA-SCARS, transmission electron microscopy (TEM), senescence-associated secretory phenotype (SASP)

## Abstract

Irreparable double-strand breaks (DSBs) in response to ionizing radiation (IR) trigger prolonged DNA damage response (DDR) and induce premature senescence. Profound chromatin reorganization with formation of senescence-associated heterochromatin foci (SAHF) is an essential epigenetic mechanism for controlling the senescence-associated secretory phenotype (SASP). To decipher molecular mechanisms provoking continuous DDR leading to premature senescence, radiation-induced DSBs (53BP1-foci) and dynamics of histone variant H2A.J incorporation were analyzed together with chromatin re-modeling in human fibroblasts after IR exposure. High-resolution imaging by transmission electron microscopy revealed that persisting 53BP1-foci developed into DNA segments with chromatin alterations reinforcing senescence (DNA-SCARS), consistently located at the periphery of SAHFs. Quantitative immunogold-analysis by electron microscopy revealed that H2A.J, steadily co-localizing with 53BP1, is increasingly incorporated into DNA-SCARS during senescence progression. Strikingly, shRNA-mediated H2A.J depletion in fibroblasts modified senescence-associated chromatin re-structuring and abolished SASP, thereby shutting down the production of inflammatory mediators. These findings provide mechanistic insights into biological phenomena of SASP and suggest that H2A.J inhibition could ablate SASP, without affecting the senescence-associated growth arrest.

## 1. Introduction

Cellular senescence is a critical hallmark of aging. Senescence is a stable proliferation arrest characterized by profound changes in cellular morphology and metabolism as well as by extensive chromatin reorganization in the nucleus. Cellular senescence was first discovered in cultured fibroblast cells, where prolonged passaging and replicative exhaustion led to growth arrest due to critically short telomeres [1]. Senescence was eventually also observed in cells subjected to a variety of stressors, including acute DNA damage, oxidative stress, or oncogene induction. Senescent cells are characterized by cell-autonomous proliferative arrest with resistance to mitogenic signals, expression of senescence-associated beta-galactosidase (SA-β-Gal), formation of senescence-associated heterochromatic foci (SAHF), and release of inflammatory cytokines and chemokines, known as the senescence-associated secretory phenotype (SASP) [2].

Normal cells undergo senescence in response to severe or irreparable DNA damage. Among the genome damage induced by ionizing radiation (IR), double-strand breaks (DSBs) represent the most biologically deleterious type of DNA lesion [3]. To tackle these potentially lethally damaging lesions, cells have evolved orchestrated and conserved mechanisms known collectively as the DNA damage response (DDR) [4]. The DDR coordinates cellular DSB repair activities immediately after the damage is detected, and multiple DSB repair proteins are recruited to DSB sites within seconds after IR exposure, forming radiation-induced foci detectable by immunofluorescence microscopy (IFM). Of particular importance in this context is the recruitment of 53BP1 to DSB-flanking chromatin [5], supposed to stabilize the nucleosomal barrier to inhibit DNA end-resection [6]. Most radiation-induced DSBs are repairable and these transient 53BP1-foci typically resolve within 24 h. However, severe or irreparable DNA damage, such as complex DSBs, cause persistent 53BP1-foci, detectable for days and weeks after their formation, suggesting that DSB repair mechanisms are incapable of resolving these lesions [7,8,9,10,11]. The focal accumulation of DNA repair factors around DNA lesions is a characteristic feature of the cellular response to DNA damage.

One striking characteristic of senescent cells is the large-scale reorganization of their chromatin, exemplified by the formation of SAHFs [12,13]. SAHFs are microscopically discernible as condensed heterochromatic structures and have been widely used as a senescence marker [14]. Previous studies indicate that chromatin restructuring at the onset of senescence implies the formation of so-called *‘DNA segments with chromatin alterations reinforcing senescence’* (DNA-SCARS) [15]. Elevated concentrations of modified histones (e.g., γH2AX) and other proteins (e.g., 53BP1) are recruited to remodeled chromatin of DNA-SCARS. These DNA-SCARS are supposed to trigger constitutive DNA damage signaling, prolonged p53-dependent growth arrest, and eventually an irreversible senescence arrest [16]. However, the precise relationship between radiation-induced DNA breaks, development of DNA-SCARS, and formation of SAHFs with higher-order chromatin rearrangements is currently unknown.

The fundamental repeating unit of chromatin is the nucleosome. The nucleosome core particle comprises 147 base pairs of DNA wrapped around the core histone octamer, containing two copies each of the canonical histone proteins H2A, H2B, H3, and H4. The linker histone H1 and non-histone proteins participate in the dynamic regulation of chromatin compaction within the nucleus. Post-translational histone modifications can directly modulate chromatin structure by altering the charge of histones, and are correlated with open/closed or active/repressed chromatin states [17]. Additional diversity is provided by the incorporation of histone variants into chromatin [18]. Histone variants can profoundly change chromatin properties by modulating nucleosome stability and function, which may affect their interaction with chromatin remodelers and modifiers [19]. The deposition of canonical histones is coupled to DNA synthesis, whereby canonical histones assemble into nucleosomes behind replication forks and at sites of DNA repair. Incorporation of histone variants, by contrast, occurs throughout the cell cycle and is independent of DNA synthesis. In non-cycling senescent cells, canonical histone production declines (due to reduced synthesis and high turnover) and variant histones tend to accumulate [20]. Histone modifications on canonical and variant histones may further affect the level of chromatin compaction, and may entail an imbalance of activating and repressive histone marks, thereby regulating gene expression [19,20,21].

In previous studies, it could be shown that the histone variant H2A.J accumulates in human fibroblasts during replicative and oncogene-induced senescence and affects inflammatory gene expression of senescent cells [22]. Recent work demonstrates the functional importance of H2A.J-specific residues and potential mechanisms for its function in promoting inflammatory gene expression in senescence. H2A.J accumulation contributes to weakening the association of histone H1 to chromatin and increasing its turnover. Decreased H1 in senescence is correlated with increased expression of some repeated DNA sequences, increased expression of STAT/IRF transcription factors, and transcriptional activation of interferon-stimulated genes [23].

A better understanding of the molecular mechanisms that drive senescence and SASP expression may help to unravel the complicated role of senescence in age-related diseases, including cancer. Depending on the cell type and initiating event, the SASP can be tumor suppressive in normal cells and favorable for tumor treatment response by enforcing arrest and promoting immune clearance of damaged cells. Alternatively, some senescent cells can secrete factors that create an immunosuppressive environment that promotes tumor initiation and progression. The reduction of SASP might provide therapeutic opportunities that could complement cancer therapy to limit tumor progression and metastasis [24].

Using IR to initiate DNA damage-induced senescence in human fibroblasts, we investigated the spatiotemporal dynamics of transient and persistent 53BP1-foci at DSBs sites, the formation of DNA-SCARS, and their impact on establishing and maintaining senescence-associated phenotypes. Here, we show that H2A.J is incorporated predominantly in radiation-induced DNA-SCARS during senescence progression and triggers decisively the development of the SASP.

## 2. Results

### 2.1. Short-Term H2A.J Accumulation Following IR Exposure

Previous studies showed that the H2A.J histone variant is present at very low levels in proliferating human fibroblasts but accumulated significantly in the chromatin of fibroblasts in replicative or oncogene-induced senescence [22]. Here, we analyzed primary (WI-38 wild-type, WT) and genetically modified human fibroblasts after IR exposure to investigate the role of H2A.J accumulation during acute and chronic DDR. As previously described, lentiviral shRNA constructs were used to knock-down H2A.J in immortalized fibroblasts (WI-38hTERT/ptet-on-sh3-H2AFJ knock-down, KD) compared to non-targeted controls (WI-38hTERT/ptet-on-sh-no-target, NT). These KD and NT fibroblasts retained normal morphological phenotypes, but their ectopic telomerase expression prevented telomere shortening-dependent replicative senescence and therefore extended their lifespan. H2A.J knock-down had no obvious effect on the efficiency with which they entered into and maintained the senescent state after etoposide treatment, nor did H2A.J depletion significantly impact cell morphology, DNA compaction, or distribution of γH2AX-foci in etoposide-induced senescence [22].

**Figure 1:**
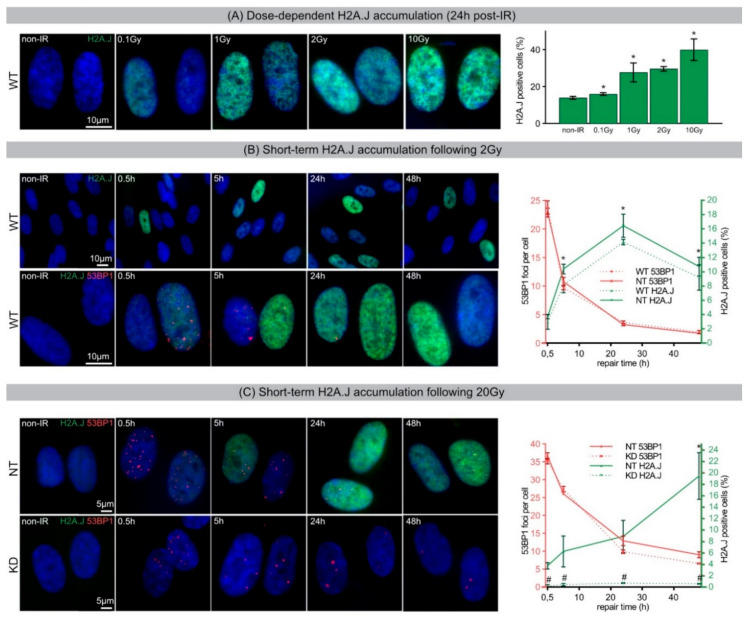
(**A**) Dose-dependent H2A.J accumulation (24 h post-IR). IFM micrographs of H2A.J staining in WT fibroblasts following IR exposure with varying doses (0.1, 1, 2, 10 Gy; 24 h post-IR) and accompanying quantification of H2A.J+ cells. (**B**) Short-term H2A.J accumulation following 2 Gy. IFM micrographs of WT fibroblasts show an acute increase of H2A.J+ cells following 2 Gy (upper panel) and the accumulation of H2A.J combined with 53BP1-foci (lower panel). Right graph shows quantification of 53BP1-foci and H2A.J+ cells in WT and NT fibroblasts (non-IR controls subtracted). (**C**) Short-term H2A.J accumulation following 20 Gy. IFM micrograph of NT and KD fibroblasts show the accumulation of H2A.J+ cells and 53BP1-foci following 20 Gy. Adjacent graph shows quantification of these markers (non-IR controls subtracted). Data are presented as mean of three experiments ±SE. * significant statistical difference to non-IR control (*p* < 0.05); ^#^ significant statistical difference to equivalent dose and time-point in NT fibroblasts (*p* < 0.05).

To analyze the relationship between the amount of radiation-induced DNA damage and H2A.J expression, confluent WT fibroblasts were irradiated with single doses between 0.1 and 10 Gy (X-rays), and 24 h later, cells were fixed and subjected to immunofluorescence staining for H2A.J. During this acute DDR, a dose-dependent increase of pan-nuclear H2A.J staining intensity in WT fibroblasts was observed (Figure 1A, left panel). Quantification of H2A.J+ cells showed a steady significant increase from ~10% in non-irradiated (0 Gy) to nearly ~40% in irradiated WT fibroblasts (10 Gy) (Figure 1A, right panel). Even low doses of IR (0.1 Gy) revealed slight but significantly increased numbers of H2A.J+ fibroblasts.

Immunostaining of 53BP1-foci allows sensitive monitoring of DSB induction and repair after IR exposure. To analyze DSB repair kinetics in combination with H2A.J accumulation, double-staining for 53BP1 and H2A.J was undertaken and, in both WT and NT fibroblasts, kinetics of 53BP1-foci formation and disappearance correlates with the induction and repair of DSB induced by 2 Gy (Figure 1B, left panel). At 0.5 h post-IR, the highest numbers of 53BP1-foci (~24 foci/cell) were observed, corresponding to initial DSB damage. Subsequently, foci numbers declined within 24 h due to DSB repair and only few residual foci remained 48 h post-IR (~2 foci/cell) (Figure 1B, right panel). Only a low number of H2A.J+ cells were observed 0.5 h post-IR (~3%), with the highest numbers of H2A.J+ fibroblasts found 24 h post-IR (~14–16%). At 48 h post-IR, the percentage of H2A.J+ fibroblasts decreased to ~10% (Figure 1B, right panel). These findings show that during acute DDR with moderate doses, H2A.J accumulation peaks 24 h post-IR and then gradually declines. This reversible chromatin incorporation of H2A.J correlates with transient radiation-induced cell-cycle arrest. Notably, NT fibroblasts (as non-targeted controls) showed no difference in their 53BP1-foci kinetics compared to primary WT fibroblasts.

Subsequently, acute DDR after high-dose IR with 20 Gy was analyzed by investigating 53BP1-foci formation and resolution kinetics. Maximum accumulation of 53BP1-foci in NT and KD fibroblasts reached~36 foci/cell at 0.5 h post-IR, reflecting initial DSB damage. Subsequently foci numbers decreased gradually to ~7–9 foci/cell 48 h post-IR. In NT fibroblasts, the number of H2A.J+ cells increased from ~4% at 0.5 h post-IR to almost ~20% at 48 h post-IR. This rising H2A.J incorporation correlates with onset of DNA-damage-induced premature senescence. In KD fibroblasts, by contrast, H2A.J accumulation was suppressed despite high levels of acute and persistent radiation-induced DNA damage. Chiefly, these findings indicate that depletion of H2A.J did not influence the kinetics of 53BP1-foci formation and resolution during acute DDR.

### 2.2. Long-Term H2A.J Accumulation Following IR and ETO Exposure

**Figure 2:**
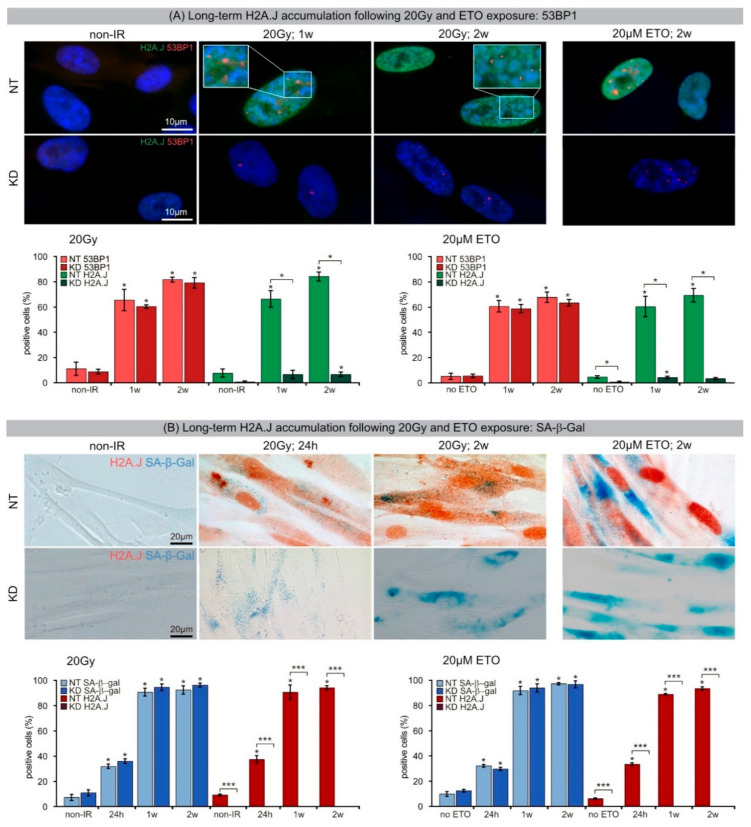
(**A**) Long-term H2A.J accumulation following IR and ETO exposure: 53BP1. IFM micrographs show H2A.J and 53BP1 staining of NT and KD fibroblasts following 20 Gy (1 and 2 weeks post-IR) or ETO exposure (2 weeks post-ETO), compared to non-exposed controls. Quantification of H2A.J+ and 53BP1+ cells 1 and 2 weeks following IR or ETO exposure, compared to non-exposed controls. (**B**) Long-term H2A.J accumulation following IR and ETO exposure: SA-β-Gal. IHC micrograph show H2A.J and SA-β-Gal staining of NT and KD 24 h and 2 weeks following IR (20 Gy) or ETO exposure, compared to non-exposed controls. Quantification of H2A.J and SA-β-Gal cells 24 h, 1 week and 2 weeks following IR or ETO exposure compared to non-exposed controls. Data are presented as mean of three experiments ±SE. Significant statistical difference compared to non-irradiated controls (marked by asterisks alone) or between NT and KD cells (asterisks with square brackets): * *p* < 0.05; *** *p* < 0.001.

To investigate possible further roles of H2A.J in DDR and investigate the effects of different DNA-damaging agents, long-term H2A.J accumulation during radiation- and etoposide-induced premature senescence was examined. Confluent NT and KD fibroblasts were irradiated with 20 Gy or incubated with 20 µM Etoposide (ETO), and after time intervals of 1 or 2 weeks, cells were fixed and stained for H2A.J and 53BP1. Through IFM, formation of condensed chromatin areas, namely SAHFs, could be observed in NT and KD fibroblasts. These striking SAHFs were accompanied by residual 53BP1-foci (red), located mainly at the periphery of SAHFs (Figure 2A, upper panel, insets). While NT fibroblasts showed intense pan-nuclear staining for H2A.J 1 and 2 weeks (w) after IR or ETO exposure, KD fibroblasts had no detectable H2A.J staining, independent of DNA-damage-inducing agent (Figure 2A, upper panel). Notably, both NT and KD fibroblasts formed SAHFs after IR or ETO exposure, indicating progression to DNA-damage-induced premature senescence. After 20 Gy, NT and KD fibroblasts revealed similar and relatively high percentages of 53BP1+ cells, with ~60–65% 1 w post-IR and ~78–82% 2 w post-IR, indicating an increase of persistent 53BP1-foci during the progression of radiation-induced senescence (Figure 2A, lower panel). However, while NT fibroblasts showed increasing proportions of H2A.J+ cells, with ~66% 1 w post-IR and ~84% 2 w post-IR, H2A.J depletion in KD fibroblasts led to consistent inherently low numbers of H2A.J+ cells (~6% at 1 and 2 weeks post-IR). Following ETO exposure, NT and KD fibroblasts revealed similarly high levels of 53BP1+ cells, with ~58–60% 1 week post-ETO and ~64–68% 2 weeks post-ETO. Whereas NT fibroblasts showed an increasing percentage of H2A.J+ cells, with 60% 1 week post-ETO and 70% at 2 weeks post-ETO, H2A.J-depleted KD fibroblasts revealed only minor numbers of H2A.J+ cells (~4% 1 week and 2 weeks post-ETO) (Figure 2A, lower panel).

SA-β-Gal is the most widely used biomarker of cellular senescence. To investigate H2A.J expression during induction and maintenance of the senescent state, double-staining for H2A.J and SA-β-Gal in NT and KD fibroblasts was established (Figure 2B). Non-exposed NT and KD fibroblasts were almost entirely negative for H2A.J and SA-β-Gal staining. After IR or ETO exposure, NT fibroblasts showed increasing staining intensity of nuclear H2A.J (brown) and cytoplasmic SA-β-Gal staining (blue), suggesting progressive entry of these cells into senescence (Figure 2B, upper panel). Scoring H2A.J+ and SA-β-Gal+ cells at different time-points following DNA damage induction confirmed a similar rise in H2A.J and SA-β-Gal in NT fibroblasts after IR and ETO exposure (H2A.J+ and SA-β-Gal+ cells: 30–36% at 24 h and 90–96% 1 and 2 weeks after IR/ETO exposure). In contrast, H2A.J-depleted KD fibroblasts showed no detectable H2A.J after IR or ETO exposure. These KD fibroblasts did, however, simultaneously exhibit an obvious increase in SA-β-Gal activity (≥90% SA-β-Gal+ cells), suggesting H2A.J is not required for entering senescence.

To test the stability of radiation-induced senescence, established markers for proliferation and cellular senescence were measured in NT and KD fibroblasts after 20 Gy (2 weeks post-IR) compared to non-IR controls. As expected, exposure to high IR doses resulted in long-term suppression of cell proliferation, induced expression of the senescence marker p21, and provoked nuclear alterations with Lamin B1 decline, thereby proving the senescent state of irradiated NT and KD fibroblasts (Supplementary Materials Figure S1).

### 2.3. Senescence-Associated Chromatin Re-Structuring Resulting in SAHF Formation

**Figure 3:**
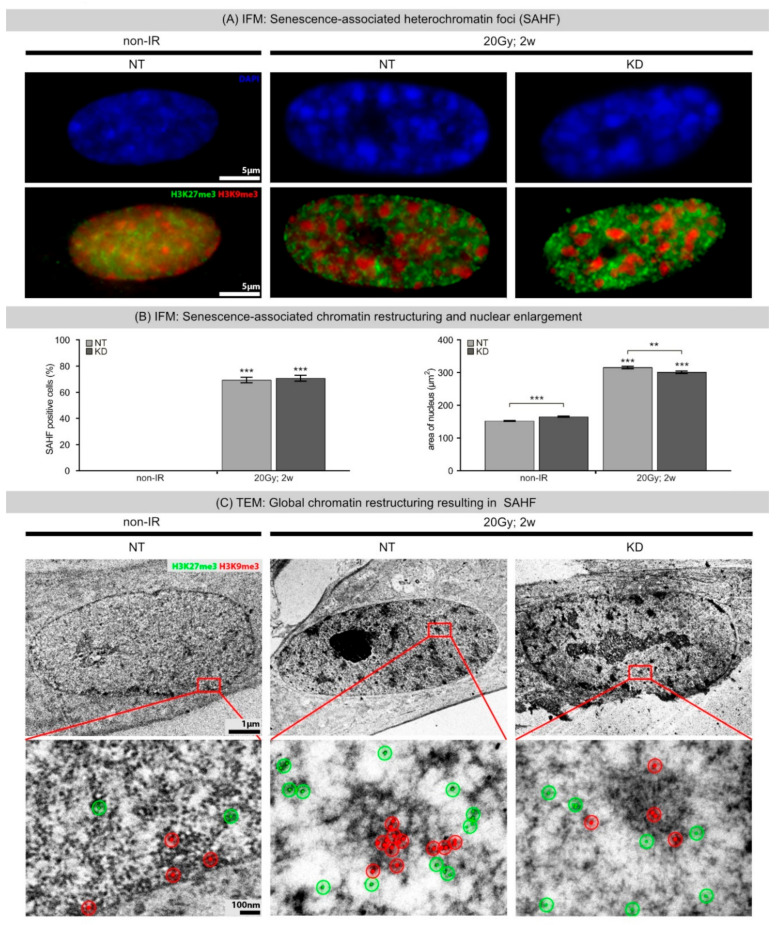
(**A**) IFM: Senescence-associated heterochromatin foci (SAHFs). IFM micrographs show DAPI, H3K9me3, and H3K27me3 staining of NT and KD fibroblasts following 20 Gy (2 weeks post-IR), compared to non-IR controls. (**B**) IFM: Senescence-associated chromatin restructuring and nuclear enlargement. Quantification of SAHF+ cells and nuclear area in NT and KD fibroblasts following 20 Gy (2 weeks post-IR), compared to non-IR controls. Data are presented as mean of three experiments ±SE. Significant statistical difference compared to non-irradiated controls (marked by asterisks alone) or between NT and KD cells (asterisks with square brackets): ** *p* < 0.01; *** *p* < 0.001. (**C**) TEM: Global chromatin re-structuring resulting in SAHFs. TEM micrographs show chromatin structure with immunogold-labeling for H3K9me3 (10-nm beads, red) and H3K27me3 (6-nm beads, green) of NT and KD fibroblasts following IR (20 Gy, 2 w post-IR), compared to non-IR controls. Red marked areas are shown with higher magnification. To aid visualization of gold-beads, red and green overlays were added, and bead clusters encircled.

Senescent cells display extensive chromatin restructuring with the formation of SAHFs. While non-irradiated NT fibroblasts exhibited only weak heterochromatin domains in DAPI-stained nuclei, irradiated NT and KD fibroblasts revealed formation of prominent DAPI-dense SAHFs during progression towards radiation-induced senescence (Figure 3A, upper panel). Further IFM studies on spatial chromatin organization showed SAHF cores enriched with constitutive heterochromatin marker H3K9me3 (red), surrounded by an outer layer of facultative heterochromatin marker H3K27me3 (green) (Figure 3A, lower panel). These findings are consistent with previous IFM studies and support the idea that SAHF formation is associated with spatial repositioning of chromatin and characteristic distribution of epigenetic marks [14]. During radiation-induced senescence progression, NT and KD fibroblasts revealed a pronounced increase of SAHF formation and nuclear enlargement at 2 weeks after 20 Gy (Figure 3B, left panel). Precise measurements by integrated evaluation software indicate that NT fibroblasts exhibited an even slightly aggravated enlargement of nuclei compared to KD cells (NT: 316.3 ± 4.0 µm^2^, KD: 301.1 ± 4.4 µm^2^) (Figure 3B, right panel). To characterize chromatin ultrastructure of SAHFs and their histone modification patterns, transmission electron microscopy (TEM) was used in combination with immunogold labeling (Figure 3C). Standard TEM preparation coupled with heavy-metal staining permits direct visualization of different chromatin density levels, producing heavily stained heterochromatin and lightly stained euchromatin. Accordingly, TEM micrographs provide high-resolution information on chromatin architecture within entire nuclei (Figure 3C, upper panel). While non-irradiated NT and KD fibroblasts showed largely homogenous chromatin organization (with slightly enhanced staining of nuclear membranes, and nucleoli), senescent NT and KD fibroblasts revealed patchy chromatin structures with multiple dense heterochromatin domains. Within the nucleus, chromatin is organized and compacted through the association of histones and their histone modifications, which together regulate the accessibility of different gene sequences, thus controlling genomic activity. To obtain a refined characterization of SAHF structures, immunogold staining for the precise localization of histone modifications in the chromatin context was established. Using gold particles of different sizes, our TEM analysis revealed H3K9me3-enriched domains (6-nm beads, red) correlated with highly compact inaccessible HC domains likely repressive to transcription (SAHF core). In contrast, H3K27me3-enriched segments (10-nm beads, green) were localized to more dispersed accessible chromatin at boundary regions of SAHFs. Collectively, correlation between these IFM and TEM results emphasize that clearly structured chromatin densities are enriched with certain histone modifications. These findings support the idea that SAHF formation may lead to spatial segregation of distinct chromatin states, and thus may contribute to the maintenance and stability of the SASP gene expression profile for both active and repressed genes.

### 2.4. DNA-SCARS Identification, Accumulation, and Characterization

**Figure 4:**
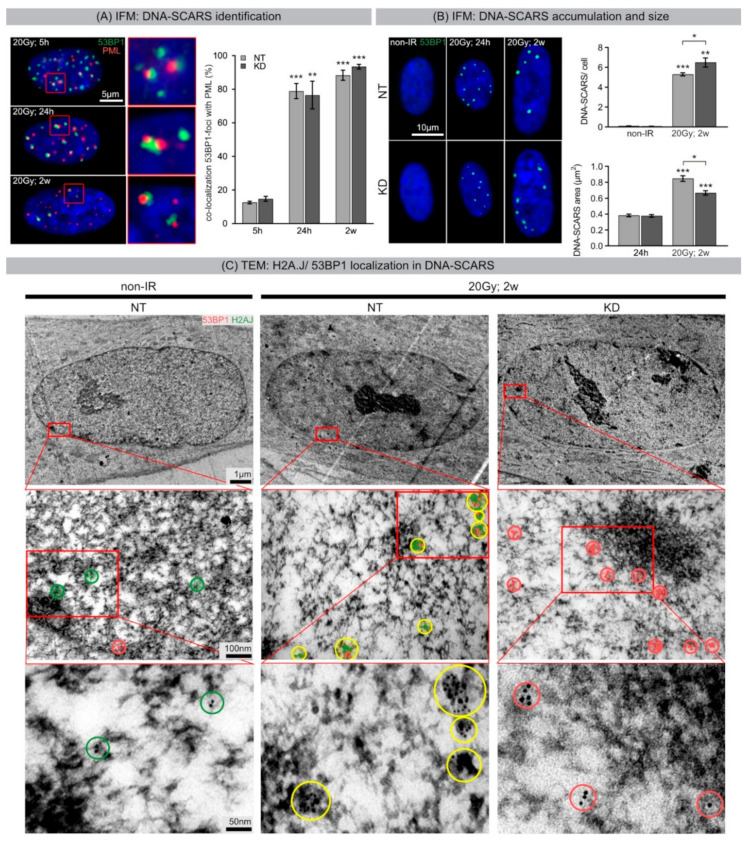
(**A**) IFM: DNA-SCARS identification. IFM micrographs of 53BP1 and PML staining of NT fibroblasts following 20 Gy (5 h, 24 h, and 2 weeks post-IR). Quantification of 53BP1-foci co-localizing with PML foci to detect acute DNA-repair foci and persistent DNA-SCARS in NT and KD fibroblasts. (**B**) IFM: DNA-SCARS accumulation and size. IFM micrographs of DNA-SCARS in non-IR controls, and 24 h and 2 weeks following 20 Gy in NT and KD cells. Quantification of DNA-SCARS in non-IR controls and 2 weeks after 20 Gy in NT and KD fibroblasts (upper panel). Area measurements of DNA-SCARS 24 h and 2 weeks following 20 Gy (lower panel). Data are presented as mean of three experiments ±SE. Significant statistical difference compared to non-irradiated controls (marked by asterisks alone) or between NT and KD cells (asterisks with square brackets): * *p* < 0.05; ** *p* < 0.01; *** *p* < 0.001. (**C**) TEM: H2A.J/53BP1 localization in DNA-SCARS. TEM micrographs show gold-labelled H2A.J (6-nm beads) and 53BP1 (10-nm beads) in NT and KD fibroblasts following 20 Gy (2 weeks post-IR), compared to non-IR controls. Red marked areas are shown with higher magnification; to aid visualization of gold-beads, green and red overlays were added, and bead clusters encircled (green: H2A.J only; red: 53BP1 only; yellow: co-localization of H2A.J and 53BP1).

An additional factor thought to play an essential role in premature radiation-induced senescence are DNA-SCARS. These chromatin alterations form after exposure to IR doses high enough to induce irreparable DNA damage, resulting in persistent DDR activation. DNA-SCARS are known to associate with a subfraction of PML nuclear bodies (PML-NBs) and trigger progressive changes in chromatin architecture [5]. Previous studies have shown that the number of PML-NBs increases in response to genomic stress from both endogenous and exogenous origins. Therefore, we quantified the PML-NB in non-irradiated and 20 Gy irradiated NT and KD fibroblasts 5 h, 24 h, and 2 weeks following IR exposure. We observed a significant rise in PML-NB in NT and KD fibroblasts following 20 Gy IR but with no significant difference in PML-NB accumulation levels between the two cell lines (Supplementary Materials Figure S3). High levels of persistent 53BP1-foci were observed at later time-points after 20 Gy IR (Figure 1C: 24 h, 48 h, Figure 2A: 1 week, 2 weeks), and thus further classification was carried out through double-staining of 53BP1 and PML (Figure 4A). While only ~13% of 53BP1-foci were associated with PML-NBs during the active DNA repair process at 5 h after 20 Gy, this proportion of co-localizing 53BP1/PML-foci significantly increased to ~78% at 24 h and ~90% at 2 weeks post-IR, with no significant difference between NT and KD fibroblasts (Figure 4A). These findings confirm that persisting 53BP1-foci reflect DNA-SCARS. While NT and KD fibroblasts showed negligible levels in non-IR controls, the number of DNA-SCARS significantly increased at 2 weeks after 20 Gy in NT and KD fibroblasts (NT: ~0.1 to ~5.3 foci/cell; KD: ~0.07 to ~6.5 foci/cell). Notably, after IR exposure, H2A.J KO fibroblasts showed significantly greater numbers of DNA-SCARS per cell than their NT counterparts (Figure 4B, upper panel). Additionally, the measured areas revealed marked expansions of DNA-SCARS between 24 h and 2 weeks after 20 Gy, in both NT and KD cells (NT: ~0.383 µm^2^ to ~0.843 µm^2^; KD: ~0.380 µm^2^ to ~0.667 µm^2^) (Figure 4B, lower panel). However, after IR exposure, KD fibroblasts showed significantly higher numbers of DNA-SCARS but with significantly smaller areas compared to NT fibroblasts, suggesting that H2A.J may affect the structural formation of DNA-SCARS.

Using immunogold-labeling for TEM, we analyzed the spatial distribution of H2A.J and 53BP1 to further investigate their functional role during senescence-associated chromatin restructuring. Cell monolayers of NT and KD fibroblasts were exposed to 20 Gy, and 2 weeks later, cells were fixed, embedded, sectioned, and immunogold-labeled for H2A.J and 53BP1. TEM micrographs of non-irradiated NT and KD fibroblasts show mostly homogenous chromatin structures (Figure 4C, left panels). While non-irradiated NT fibroblasts exhibited low levels of H2A.J (marked green), KD fibroblasts exhibited almost no H2A.J. Moreover, non-irradiated NT and KD fibroblasts revealed only low amounts of 53BP1 (marked red), evenly distributed throughout the entire nucleus (Figure 4C, left panels). Following 20 Gy exposure, NT and KD fibroblasts underwent dramatic chromatin alterations during senescence progression, resulting in the formation of SAHFs. Highly compact SAHF cores were surrounded by irregularly shaped substructures in more decondensed chromatin regions, likely reflecting DNA-SCARS (Supplementary Materials Figure S2). Remarkably, TEM analysis of senescent NT fibroblasts revealed large clusters of H2A.J constantly co-localizing with 53BP1 at DNA-SCARS in the periphery of SAHFs. This phenomenon was not evident at the low-resolution light microscopy level but detectable only through the markedly increased resolution attained by TEM. Senescent KD fibroblasts, by contrast, showed high amounts of 53BP1 clusters in DNA-SCARS but without any H2A.J co-localization.

### 2.5. Quantification of H2A.J and 53BP1 in DNA-SCARS

**Figure 5:**
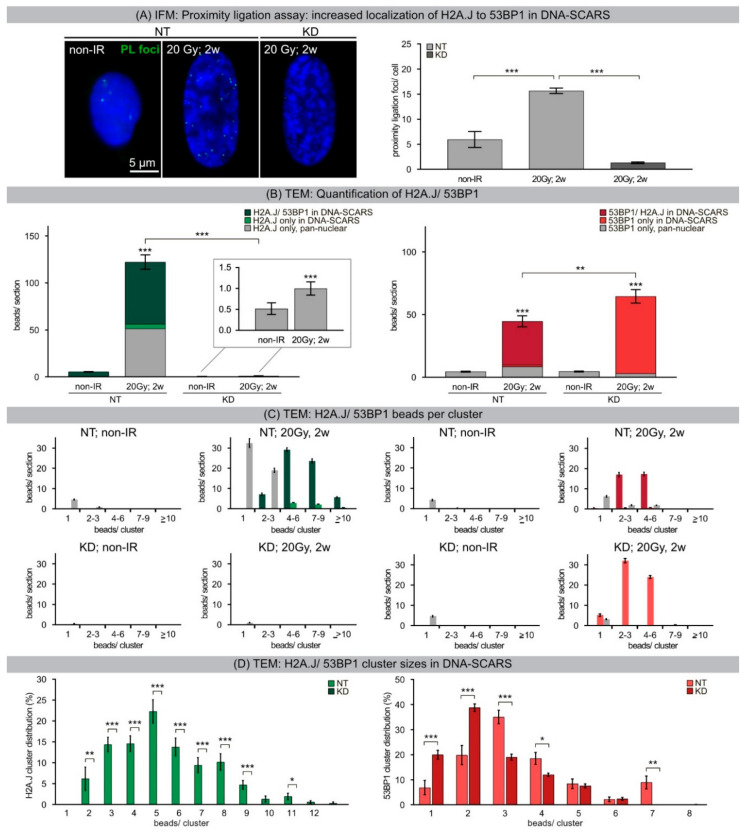
(**A**) IFM: Proximity ligation assay: increased localization of H2A.J to 53BP1 in DNA-SCARS. IFM micrograph of proximity ligation foci generated between 53BP1 and H2A.J in NT and KD fibroblasts in non-IR control and 2 weeks after 20 Gy IR. Quantification of PLA foci in NT and KD fibroblasts in non-IR control and 2 weeks after 20 Gy. Data are presented as mean of three experiments ±SE. (**B**) TEM: Quantification of H2A.J/53BP1 beads. H2A.J- and 53BP1-labeled gold-beads were quantified in 25 nuclear sections of NT and KD fibroblasts after 20 Gy (2 w post-IR), compared to non-IR controls. Different colors indicate potential co-localization between H2A.J/53BP1 and spatial localization inside and outside DNA-SCARS. (**C**) TEM: Quantification of H2A.J/53BP1 beads per cluster. For H2A.J and 53BP1, the number of beads was quantified for different cluster sizes (1, 2–3, 4–6, 7–9, ≥10 beads/cluster) in 25 nuclear sections of NT and KD fibroblasts following 20 Gy (2 w post-IR), compared to non-IR controls. (green: H2A.J/53BP1 co-localization inside DNA-SCARS; light green: H2A.J alone inside DNA-SCARS; grey H2A.J alone outside DNA-SCARS; dark red: 53BP1/H2A.J co-localization inside DNA-SCARS; red: 53BP1 alone inside DNA-SCARS; grey: 53BP1 outside DNA-SCARS). (**D**) TEM: H2A.J/53BP1 cluster sizes within DNA-SCARS. Quantification of H2A.J and 53BP1 cluster size distribution as percentage of total beads within DNA-SCARS. All TEM data are presented as mean 25 nuclear sections ±SE. Significant statistical difference compared to non-irradiated controls (marked by asterisks alone) and between NT and KD cells (asterisks with square brackets): * *p* < 0.05; ** *p* < 0.01; *** *p* < 0.001.

Unlike TEM, the resolution of IFM is insufficient to visualize the accumulation of H2A.J at DNA-SCARS in proximity to 53BP1. To combat this technical issue, a proximity ligation assay between H2A.J and 53BP1 was performed such that a fluorescent signal would only be emitted if the two molecules lay within 40 nm or less of one another (Figure 5A, left panel). Quantification of these proximity ligation assay (PLA) foci in NT fibroblasts revealed a dramatic increase in quantity from non-irradiated (~6 foci/cell) to 20-Gy-irradiated fibroblasts 2 weeks post-IR (15.7 foci/cell) (Figure 5A, right panel). Moreover, these PLA foci were consistently located at the margins of SAHFs. In contrast, KD fibroblasts irradiated with 20 Gy and analyzed 2 weeks after IR showed only ~1.3 foci/cell, reflecting the absence of H2A.J. To further support these findings, TEM was used for quantitative analysis of H2A.J and 53BP1 by labeling the proteins with gold-beads of different sizes and counting these in 25 nuclear sections, whilst simultaneously noting their localization within the chromatin context (Figure 4C and Figure 5B). Non-irradiated NT fibroblasts revealed few single H2A.J beads throughout the entire nuclear section (5 beads/section). After 20 Gy exposure, senescent NT fibroblasts exhibited extremely high H2A.J levels (123 beads/section), whereby the majority of beads were localized within DNA-SCARS (71 beads/section; ~58%) (Figure 5B, left panel). Further sub-analysis of the H2A.J distribution showed large clusters of 4–12 beads within DNA-SCARS (representing only 3% of the total nuclear section area), with only single beads or small clusters (≤3 beads/cluster) in the remaining section area (97% of the nuclear section area) (Figure 5C, left upper panel). Further analysis revealed ~94% of all H2A.J clusters in DNA-SCARS were larger clusters containing three beads or more (Figure 5D). KD fibroblasts revealed almost no H2A.J beads or clusters, independent of IR exposure (Figure 5B–D).

While non-irradiated NT and KD fibroblasts revealed only few single 53BP1 beads, this damage-associated factor revealed large 53BP1 clusters within DNA-SCARS of senescent NT and KD fibroblasts (Figure 5B, right panel). Significantly, senescent NT fibroblasts showed large 53BP1-clusters (Figure 5C, right upper panel) co-localizing with H2A.J in DNA-SCARS (35 beads/section; ~78%) (Figure 5B, right panel), suggesting that H2A.J accumulates at damaged chromatin. Senescent KD fibroblasts with knocked-down H2A.J expression revealed significantly more 53BP1 gold-beads, which, however, also predominantly localized within DNA-SCARS (62 beads/section, ~95%) (Figure 5B, right panel), but with greater amounts of smaller clusters (Figure 5C, right lower panel). Comparison of the cluster distribution within DNA-SCARS revealed the majority of 53BP1 clusters in KD fibroblasts (~59%) contained two beads or less compared to NT fibroblasts in which this number of clusters with ≤2 beads reached only ~26% (Figure 5D, right panel). Overall, NT fibroblasts demonstrated higher levels of larger 53BP1 clusters than KD fibroblasts with significant differences in three- (NT: ~35%; KD: ~19%), 4- (NT: ~18%; KD: ~12%) and seven-bead clusters (NT: ~9%; KD: 0%) respectively. These quantitative TEM results indicate that H2A.J is preferentially integrated into damaged chromatin at DNA-SCARS. KD fibroblasts, entering the senescent state after 20 Gy exposure, also revealed DNA-SCARS, but these DNA-SCARS did not contain any H2A.J. Supporting the results of IFM analysis, DNA-SCARS of senescent KD fibroblasts revealed higher 53BP1 deposition, potentially to compensate for lack of H2A.J during chromatin remodeling. In summary, our quantitative TEM results indicate that H2A.J is preferentially incorporated at damaged chromatin regions of DNA-SCARS in the periphery of SAHFs. Significantly, depletion of H2A.J did not prevent the formation of DNA-SCARS or SAHFs, nor the cellular stress response of premature senescence. However, H2A.J depletion influenced in particular the modality of 53BP1 accumulation within DNA-SCARS, and therefore the fine-tuning of chromatin restructuring.

### 2.6. Senescence-Associated Secretory Phenotype Analysis

**Figure 6:**
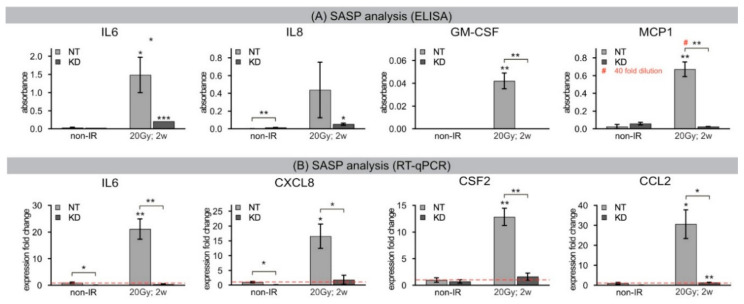
(**A**) ELISA: SASP analysis. Quantification of SASP factors in conditioned medium of NT and KD fibroblasts analyzed 2 weeks after 20 Gy (2 weeks post-IR), compared to non-IR controls. # For MCP1 analysis NT 20 Gy, 2 weeks post-IR sample was diluted by factor 40 (10,000 cells/mL of conditioned medium) to ensure results lie within the linear range of the assay. (**B**) RT-qPCR: SASP analysis. Quantification of expression levels of equivalent SASP genes analyzed 2 weeks after 20 Gy (2 weeks post-IR), compared to non-IR controls. Results were normalized to GAPDH and to corresponding gene expression levels in non-IR NT controls. Red dotted line indicates the mRNA expression of non-irradiated NT cells. Significant statistical difference compared to non-irradiated controls (marked by asterisks alone) or between NT and KD cells (asterisks with square brackets): * *p* < 0.05; ** *p* < 0.01.

Senescent cells remain viable in culture for long intervals and develop SASP with secretion of numerous cytokines and growth factors. To analyze the functional role of H2A.J for triggering SASP, we used enzyme-linked immunosorbent assay (ELISA) to investigate prominent senescence-associated factors secreted by human fibroblasts: the pleiotropic proinflammatory cytokines interleukin-6 (IL6) and interleukin-8 (IL8), granulocyte-macrophage colony-stimulating factor (GM-CSF), and monocyte chemoattractant protein-1 (MCP1). Results showed that non-irradiated NT and KD fibroblasts expressed low levels of these common SASP factors, and hence no senescence-messaging secretome (Figure 6A). NT fibroblasts in radiation-induced senescence, by contrast, secreted high levels of IL6, IL8, GM-CSF, and MCP1, demonstrating that SASP components were significantly increased between non-senescent and senescent states in NT fibroblasts (Figure 6A). Dependent on the SASP factor analyzed, 20–300-fold changes in senescence-messaging secretome were measured. Strikingly, KD fibroblasts revealed distinctly lower expression levels of these SASP factors in IR-induced senescence with only minor senescence-associated changes (Figure 6A). These findings indicate that despite persistent DNA-damage-associated senescence, H2A.J depletion attenuates the SASP by downregulating these soluble signaling factors. Subsequently, reverse-transcriptase quantitative polymerase-chain-reaction (RT-qPCR) was performed to monitor relative gene expression of these SASP factors during radiation-induced senescence. As expected, non-irradiated NT and KD fibroblasts contained low levels of RNA coding for these SASP components (Figure 6B). Gene expression analysis of NT fibroblasts after senescence-inducing IR revealed significantly increased mRNA expression of IL6, CXCL8, CSF2, and CCL2 with ~10–30-fold increases (Figure 6B). These findings confirm the robust induction of senescence-associated genes during radiation-induced senescence. Notably, H2A.J depletion in senescent KD fibroblasts almost abolished upregulation of these senescence-associated genes, suggesting that transcriptional induction of these SASP components depends on H2A.J (Figure 6B). Collectively, our findings indicate that gene transcription and protein expression of SASP factors are directly influenced by H2A.J and lack of H2A.J incorporation into DNA-SCARS prevents SASP development in response to senescence-inducing DNA damage.

## 3. Discussion

Cellular genomes are constantly exposed to DNA damage, such as IR, threatening not only genome stability but also the integrity of chromatin organization. The DDR coordinates DNA repair activities and chromatin dynamics following radiation-induced damage, arrests cell-cycle progression, and is the central mediator in triggering cellular senescence [15,16]. To investigate the molecular mechanisms that eventually provoke continuous DDR leading to radiation-induced senescence, we analyzed chromatin dynamics of the histone variant H2A.J during the repair of radiation-induced DSBs in human fibroblasts. Senescence is associated with profound chromatin reorganization and in particular with the formation of SAHFs, where condensed chromatin is supposed to repress the expression of proliferation-promoting genes. Using high-resolution imaging techniques, we found that persisting 53BP1 foci after DNA-damaging IR transform into DNA-SCARS, which were consistently located in peripheral regions of SAHFs. Quantitative immuno-electron microscopic analysis revealed that H2A.J co-localizes preferentially with 53BP1 at damaged chromatin, and is steadily incorporated into these radiation-induced DNA-SCARS during senescence progression. H2A.J depletion in KD fibroblasts did not affect the stability of cell-cycle arrest nor the coarser re-structuring of senescence-associated chromatin with formation of SAHFs. However, H2A.J depletion appeared to influence the nanostructure of DNA-SCARS and to suppress the expression of SASP, and thereby inhibited the chronic production of inflammatory mediators. Accordingly, the incorporation of H2A.J at damaged chromatin may directly affect the transcriptional program of senescent cells. These findings suggest that H2A.J inhibition could be a reasonable strategy to ablate the SASP without affecting the senescence-associated growth arrest.

During acute DDR, most radiation-induced 53BP1 foci resolved within 24 h post-IR, but ~10% of 53BP1 foci persist for days and weeks, particularly after high-dose exposure. The focal accumulation of DNA repair factors, including 53BP1 and PML nuclear bodies, is a key cytological signature of DDR [25,26]. Although persistent DNA damage foci have been studied by super-resolution localization microcopy [27,28,29], our knowledge of persistent DNA damage triggering SASP is still incomplete. In previous light and electron microscopic studies, we characterized the ultrastructure of radiation-induced foci in the chromatin context within the nuclei of normal human fibroblasts. By time course experiments with immuno-gold labeling of 53BP1, γH2AX, and other key repair factors, such as Ku70-Ku80, we could show that DNA repair foci are highly dynamic, with early and late repair foci exhibiting distinct compositions of repair factors and chromatin arrangements [8,30]. During acute DDR, 53BP1 accumulates on chromatin regions in the vicinity of radiation-induced DSBs. These early 53BP1-foci typically contain core components of DSB repair pathways, such as the Ku70-Ku80 heterodimer of non-homologous end-joining (NHEJ) [8]. However, persistent 53BP1 foci usually lack these key repair factors, and thus are no longer sites of active DNA damage repair. Instead, they may reflect stable chromatin alterations remaining from radiation-induced DNA lesions [8,15,16,25,26]. Notably, at the early stage of DDR, our TEM studies revealed no global chromatin restructuring, and particularly no specific chromatin domains with circumscribed H2A.J incorporation. These findings suggest that the characteristic remodeling of the nuclear architecture in senescent cells may rely on progressive chromatin maturation events after completion of DNA repair.

Recent reports emphasize the great importance of chromatin remodeling during DSB repair in response to DNA-damaging IR [3,4,31,32]. Etoposide, another widely used anticancer agent, inhibits topoisomerase activity, impairs DNA strand rejoining functions, and thereby induces single- and double-strand breaks [33]. Our results show that chromatin restoration after different DNA-damaging agents similarly involves the deposition of the H2A.J variant, which may not only replace damaged canonical histones but leave marks of damage experience. Following IR exposure with moderate doses (2 Gy), the reversible H2A.J incorporation correlated temporally with the transient growth arrest. Exposure to high doses of IR (≥10 Gy) or to high concentrations of etoposide resulted in progressive irreversible H2A.J incorporation and correlated with the senescence-associated permanent cell-cycle arrest. Significantly, H2A.J depletion had no obvious effects on DNA damage repair, nor on the induction of the senescent state with permanent cell-cycle arrest and increased SA-β-Gal expression. However, we did observe a modification of 53BP1 foci in senescent cells depleted for H2A.J; the number of 53BP1 clusters per nuclear section increased, but the 53BP1 density within these clusters was decreased. H2A.J depletion thus perturbs the normal ultrastructure of DNA-SCARS.

Histone dynamics coupled to DNA damage repair may contribute to the maintenance of epigenome integrity during the response to DNA damage. However, destabilization of nucleosomes during the DDR allows histone exchange and may result in the replacement of canonical histones with variants that carry out specialized functions. Histone variant H2A.J differs from canonical H2A protein by the presence of an SQK motif near the C-terminus and an A11V substitution in the N-terminal tail that do not impart any considerable structural alterations to nucleosomes [22]. However, the incorporation of H2A.J instead of canonical H2A may imply the disruption of original epigenetic information at damaged chromatin sites. Our findings are in line with previous studies suggesting that high-order heterochromatin formation and epigenetic remodeling of the genome can be discrete events [34]. Previous ChIP-seq studies could not show any preferential incorporation of H2A.J in the promoter regions of SASP genes [22]. Our imaging data establish a close connection between persisting 53BP1 foci later transforming into DNA-SCARS, likely reflecting remodeled chromatin at radiation-induced DNA lesions. These DNA-SCARS were consistently localized to the periphery of SAHFs, representing the main characteristic of senescence-related chromatin disorganization. SAHF formation results generally in concentric chromatin architectures, segregating H3K9me3-enriched compact cores from H3K27me3-enriched less densely packed ring structures [34]. The segregation of distinct epigenetic components into specific chromatin compartments is believed to promote marked changes in the gene expression program, such as downregulation of cell cycle genes and upregulation of SASP genes. High-resolution TEM imaging of senescent fibroblasts shows that H2A.J was incorporated preferentially in damaged chromatin of DNA-SCARS at the periphery of SAHFs. The profound chromatin reorganization during senescence progression is an essential epigenetic mechanism for controlling the different aspects of the senescence program. However, our data indicate that the incorporation of H2A.J in radiation-induced DNA-SCARS is not necessary for the establishment of SAHFs but indispensable to promote the SASP with the production of inflammatory mediators. RNA interference-mediated knock-down of H2A.J suppressed SASP expression but did not affect senescence-related chromatin reorganization nor the stability of senescence-associated cell-cycle arrest.

Further investigations are necessary to capture the dynamic process of senescence progression in response to radiation-induced DNA damage. Remodeling of chromatin during the progression of radiation-induced senescence might be associated with varying production of different SASP factors. However, our current understanding of the interdependence of chromatin structure and nuclear function in senescent cells is extremely limited. In previous studies, Contrepois et al. examined the effect of ectopic H2A.J expression in proliferating and senescent cells in which the endogenous H2AFJ mRNA was depleted. This ectopic H2A.J overexpression did not overtly affect cell proliferation, nor did it inhibit entry into senescence in response to etoposide treatment. Significantly, this ectopic H2A.J overexpression increased the expression of inflammatory genes in both senescent and proliferating cells, independent from their different chromatin structure [22].

Collectively, our results suggest that H2A.J inhibition could be a reasonable strategy to ablate the SASP, without affecting senescence-associated growth arrest. Using epigenetic mechanisms to target H2A.J incorporation could serve to uncouple the long-term deleterious effects of SASP from the beneficial effects connected to senescence-associated growth arrest [2]. Targeting proteins controlling the senescent state has many therapeutic implications. Senescent cells accumulate with age and are detrimental for tissue structure and function, and the SASP is likely the most important contributor to the negative effects of senescent cells on tissue homeostasis [35]. Our data enhances our understanding of the epigenomic regulation of the SASP and sets the stage for the development of new therapies aimed at suppressing the inflammatory component of senescence.

## 4. Experimental Procedures

### 4.1. Cell Culture

WI-38 human embryonic fibroblasts (WI-38 wild-type, WT) were obtained from the ATCC. Lentiviral shRNA constructs were used to knock-down H2A.J in immortalized fibroblasts (WI-38hTERT/ptet-on-sh3-H2AFJ knock-down, KD) and non-targeted controls (WI-38hTERT/ptet-on-sh-no-target, NT). NT and KD fibroblasts were provided by C. Mann and prepared as described previously [22]. KD shRNA targets positions 265–283 of the human H2AFJ coding sequence: 50-CGCAACGACGAGGAGTTAA-3. Fibroblasts were cultured at 5% O_2_ and 5% CO_2_ in MEM (Invitrogen, Karlsruhe, Germany) with 10% fetal bovine serum, 1 mM sodium pyruvate, 2 mM L-glutamine, 0.1 mM non-essential amino acids, and 1% penicillin/streptomycin. Then, 1 µg/mL doxycycline was added to medium for 1 week prior to experimental begin to activate sh inserts. Cells were then grown on coverslips and used once 90% confluent.

### 4.2. Radiation Exposure

Cells were exposed to ionizing radiation (IR) using the linear accelerator Artiste™ (Siemens, Munich, Germany) (6-MV photons; dose-rate 2 Gy/min). Cells were analyzed following different doses (2 Gy; 20 Gy) and at different time-points (0.5 h, 5 h 24 h, 48 h, 1 week, and 2 weeks post-IR) and compared to non-irradiated controls.

### 4.3. Etoposide Exposure

Cells were incubated with 20 µM Etoposide for the duration of experiments (1 or 2 weeks) and compared to non-exposed confluent controls.

### 4.4. IFM Analysis

Cells were fixed with 4% paraformaldehyde and permeabilized with 0.5% Triton X-100, washed with 0.1% Tween^®^-20, and incubated overnight with primary antibody (anti-H2A.J supplied by C. Mann; anti-53BP1, Merck, Darmstadt, Germany; anti-H3K27me3, anti-H3K9me3, and anti-p21, Abcam, Berlin, Germany; anti-ki67, Thermo Fisher, Waltham, MA, USA; anti-lamine B1, Proteintech, Manchester, UK; anti-PML, Santa Cruz, CA, USA) followed by Alexa-Fluor^®^488 or Alexa-Fluor^®^568 secondary antibody (Invitrogen, Karlsruhe, Germany). Subsequently, cells were mounted in VECTAshield™ mounting medium with 4′, 6-diamidino-2-phenylindole (DAPI, Vector Laboratories, Burlingame, CA, USA). Fluorescence images were captured with a Nikon-Eclipse Ni fluorescence microscope equipped with a Nikon DS-Qi2 camera (Nikon, Düsseldorf, Germany).

For evaluating H2A.J, 53BP1, SAHF, Ki67, and p21 positivity, at least 200 cells were captured for each sample (positive cells in %). For counting 53BP1 foci per cell, at least 50 foci and/or 50 cells were analyzed per sample. For identification of DNA-SCARS, 53BP1/PML co-localization was analyzed in 25 nuclei and numbers of 53BP1/PML co-localizing foci relative to total 53BP1 foci were expressed in percentages. Area of nuclei, area of 53BP1-foci, and lamine B1 fluorescence intensity (FITC signal normalized to DAPI) were measured based on their DAPI/FITC signal in 200 cells using Nikon NIS-Elements Basic Research acquisition software. Region of Interests (ROIs) were defined by appropriate fluorescence thresholds to measure the structure size accurately in an unbiased and transparent manner.

### 4.5. BrdU Labeling

To test the stability of senescence-associated growth arrest, non-irradiated (non-IR) and senescent cells (20 Gy; 2 weeks post-IR) were pulsed with 10 µmol/L 5-bromo-2′-deoxyuridine (BrdU) in culture medium for 24 h. After medium removal and several PBS washing steps, cells were fixed and permeabilized as described for standard IFM. DNA denaturation was completed through 1-h incubation in 2 M HCl succeeded by PBS washes and 2-h incubation with primary anti-BrdU antibody (Bio-Rad Laboratories, Munich, Germany) in 0.1% Tween, 1% BSA in PBS. After washes of Tween/BSA/PBS, samples were incubated with fluorescence-coupled anti-rat secondary antibody in Tween/BSA/PBS for 2 h. After PBS washes, coverslips were mounted with hard-set mounting medium containing DAPI.

### 4.6. Proximity Ligation Assay

A Duolink^®^ PLA system (DUO92002, -004, -014, -040, -049, Sigma-Aldrich, St. Louis, MO, USA) was used to detect co-localizations of H2A.J and 53BP1 molecules with spacing distances ≤40 nm, according to supplier protocol. Subsequently, samples were prepared and fixed as for standard IFM and PLA foci for 50 cells were quantified.

### 4.7. IHC Analysis (SA-β-Gal)

Following 5-min fixation with 2% paraformaldehyde and 0.2% glutaraldehyde, cells were incubated with X-Gal staining solution (AppliChem GmbH, Darmstadt, Germany) at 37 °C overnight. After a 30-s methanol incubation, dried samples were permeabilized with 0.2% Triton X-100 and washed with 1% BSA. Samples were blocked with 4% BSA for 1 h, followed by an overnight incubation with H2A.J primary antibody (anti-H2A.J supplied by C. Mann). Incubation with Dako immunoglobulin/bioatinylated secondary antibody (Agilent, Waldbronn, Germany) was followed by Vectastain ABC Peroxidase standard (Vector, Burlingame, CA, USA) and SIGMAFAST™ 3.3′ Diaminobenzidine (Merck, Darmstadt, Germany) incubations, respectively. Samples were finally mounted in Dako Faramount Mounting Medium (Agilent, Waldbronn, Germany).

### 4.8. TEM Analysis

Cells were fixed overnight using 2% paraformaldehyde and 0.05% glutaraldehyde in PBS. Samples were dehydrated in increasing ethanol concentrations and permeated overnight with LR White resin (EMS, Hatfield, PA, USA) followed by overnight embedding at 50 °C with fresh LR White resin containing LR White Accelerator (EMS, Hatfield, PA, USA). Microtome Ultracut UCT (Leica, Wetzlar, Germany) and diamond knife (Diatome, Biel, Switzerland) were implemented to acquire ultrathin sections (70 nm) picked up on pioloform-coated nickel grids and prepared for immunogold-labeling. Non-specific staining was blocked using Aurion blocking solution (Aurion, Wageningen, The Netherlands), sections were rinsed and incubated overnight with primary antibodies (anti-H2A.J supplied by C. Mann; anti-53BP1, Merck, Darmstadt, Germany; anti-H3K27me3 and anti-H3K9me3, Abcam, Berlin, Germany) at 4 °C, followed by incubation with 6- or 10-nm gold particle-conjugated secondary antibodies (Aurion, Wageningen, The Netherlands) for 1.5 h. Finally, sections were fixed with 2% glutaraldehyde and contrasted with uranyl acetate. A Tecnai Biotwin™ transmission electron microscope (FEI, Eindhoven, The Netherlands) was employed for visual analysis. For quantification, single beads and bead clusters were counted in 25 nuclear sections, additionally noting co-localizations as well as chromatin localization (hetero-/euchromatin; inside/outside DNA-SCARS).

### 4.9. ELISA Analysis

Conditioned media was isolated from three independent cultures of NT and KD fibroblasts. Media was analyzed from fibroblasts after inducing senescence by IR with 20 Gy or incubation with 20 μM etoposide for 2 weeks. Cell numbers in culture dishes were determined at the same time that media was collected and frozen at −80 °C until analysis. A Multi-Analyte ELISArray™ Kit (Qiagen, Hilden, Germany) was used to screen SASP factors (IL6, IL8, GM-CSF, MCP1) with a standard ELISA plate reader, according to supplier protocol. Conditions were optimized to ensure readings fell within the linear range of the assay for each cytokine.

### 4.10. RT-qPCR Analysis

RNA extraction was completed using TRIzol™ (Thermo Fisher Scientific, Darmstadt, Germany) and phenol/chloroform phase separation. A QuantiTect^®^ Reverse Transcription Kit (Qiagen, Hilden, Germany) was used for cDNA synthesis from RNA and a QuantiTect^®^ SYBR^®^ Green kit (Qiagen, Hilden, Germany) for subsequent quantitative PCR in Roche LightCycler II (Roche, Mannheim, Germany). Custom primers (Table 1) were manufactured by Thermo Fisher Scientific (Darmstadt, Germany). Values were normalized to *GAPDH*.

### 4.11. Statistical Analysis

All data were presented as mean ±SEM, where normally distributed data were analyzed by Student’s *T*-Test and non-parametric data were analyzed by Mann–Whitney-*U*-Test to evaluate differences between time-points and cell lines. All statistical analyses were performed by statistical software SPSS (SPSS Statistics25, IBM, Armonk, New York, NY, USA). Statistical significance was presented as * *p* < 0.05, ** *p* < 0.01, *** *p* < 0.001.

## Figures and Tables

**Table 1:** RT-qPCR primer sequences.

**CCL2**	F CAGCCAGATGCAATCAATGCC
	R TGGAATCCTGAACCCACTTCT
**CSF2**	F TCCTGAACCTGAGTAGAGACAC
	R TGCTGCTTGTAGTGGCTGG
**CXCL8**	F TTTTGCCAAGGAGTGCTAAAGA
	R AACCCTCTGCACCCAGTTTTC
**GAPDH**	F ATGGGGAAGGTGAAGGTCG
	R GGGGTCATTGATGGCAACAATA
**IL6**	F ACTCACCTCTTCAGAACGAATTG
	R CCATCTTTGGAAGGTTCAGGTTG

## Supplementary Materials

The following are available online at https://www.mdpi.com/1422-0067/21/23/9130/s1.
