# Supplementary material for: Histone Variant H2A.J Marks Persistent DNA Damage and Triggers the Secretory Phenotype in Radiation-Induced Senescence
Source: Int J Mol Sci. 2020 Nov 30;21(23):9130. doi: 10.3390/ijms21239130 (PMC7729917; doi:10.3390/ijms21239130)
Supplement: Supplementary file 1 [file ijms-21-09130-s001.pdf]

International Journal of
Molecular Sciences

Supplemental Figures

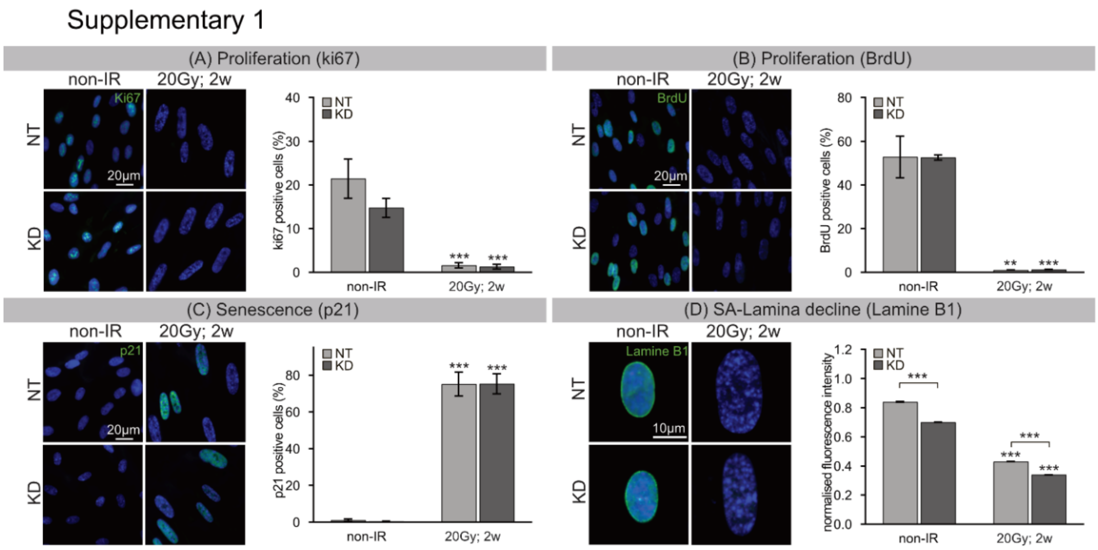

Figure S1. (A) Proliferation (ki67). IFM micrographs of ki67 staining and quantification of ki67+ cells in NT and KD fibroblasts of non-IR controls and following 20Gy (2w post-IR). **(B)** Proliferation (BrdU). IFM micrographs of NT and KD fibroblasts pulse-labelled with BrdU for 24h and quantification of BrdU+ cells in non-IR controls and following 20Gy (2w post-IR). **(C)** Senescence (p21). IFM micrographs of p21 staining and quantification of p21+ cells in NT and KD fibroblasts of non-IR controls and following 20Gy (2w post-IR). **(D)** Senescence-associated Lamina decline (Lamine B1). IFM micrographs and quantification of Lamine B1 stained NT and KD fibroblasts in non-IR controls and following 20Gy (2w post-IR). Lamine B1 was quantified through measurement fluorescence intensity per nucleus and normalized to DAPI signal. Significant statistical difference compared to non-irradiated controls (marked by asterisks alone) or between NT and KD cells (asterisks with square brackets): *p <0.05; **p<0.01; ***p<0.001.

Supplementary 2

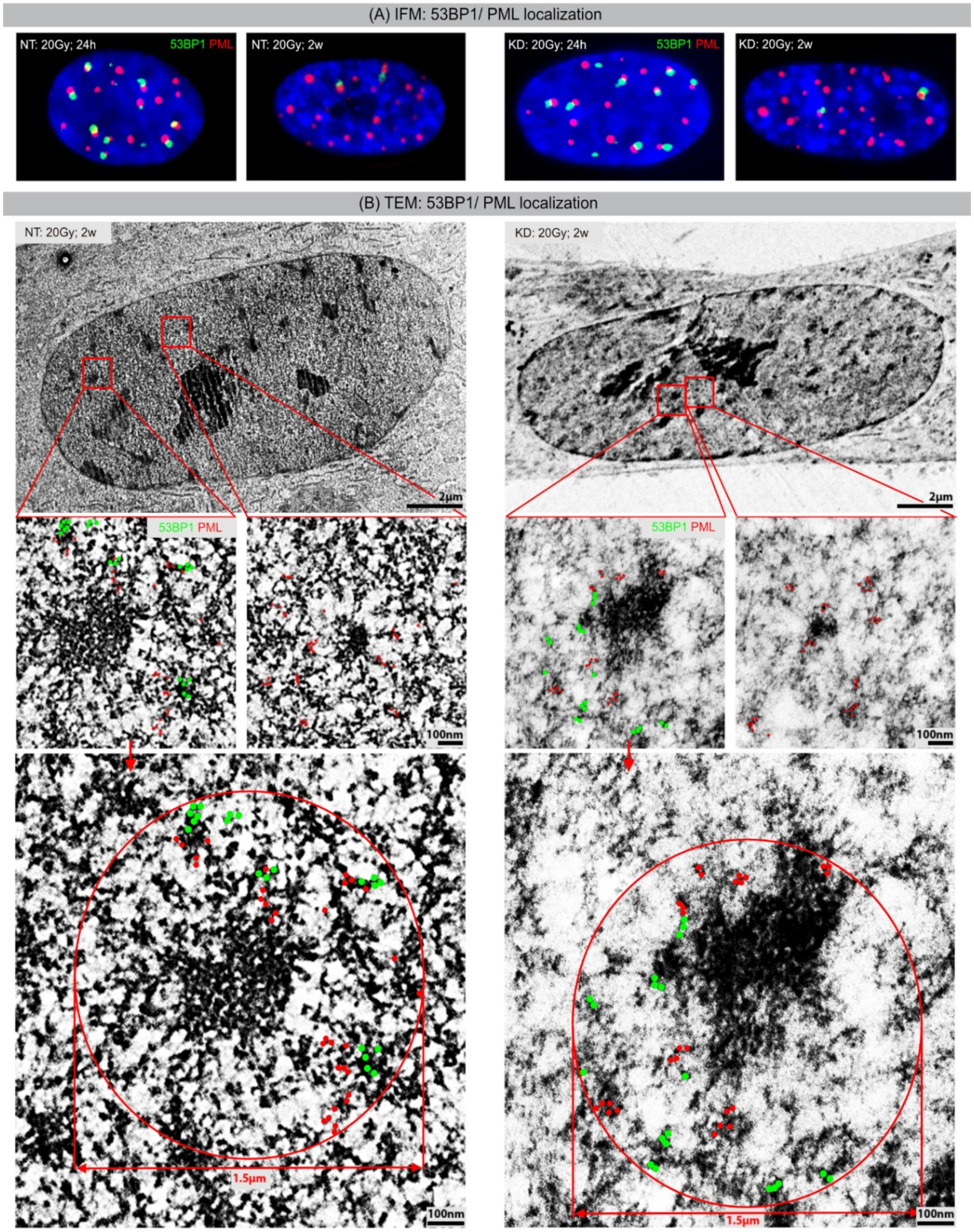

Figure S2. (A) IFM: 53BP1/PML localization**.** IFM micrographs showing increased co-localization between 53BP1 and PML with increasing time (24h, 2w) following 20Gy. **(B)** TEM: 53BP1/PML localization. TEM micrographs show gold-labelled PML (6-nm beads) and 53BP1 (10-nm beads) forming DNA-SCARS at periphery of heterochromatic SAHF in NT and KD fibroblasts following 20Gy (2w post-IR). Red marked areas shown in higher magnification; to aid visualization of gold-beads, green and red overlays were added. Red circle shows perimeter of maximum distance from SAHF in which DNA-SCARS were found and quantified.

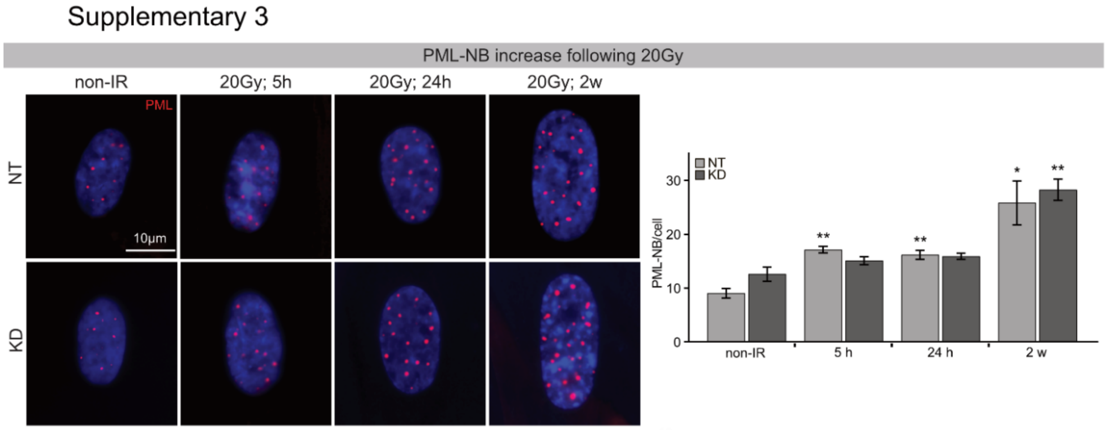

Figure S3. PML accumulation following IR. IFM micrographs of PML staining and PML foci/cell quantification showing increased PML accumulation (5h, 24h, 2w) following 20Gy. Significant statistical difference compared to non-irradiated controls: *p <0.05; **p<0.01.
